# Mechanisms of support for exclusive breastmilk expressers in the community: a scoping review

**DOI:** 10.1186/s12884-019-2667-y

**Published:** 2019-12-19

**Authors:** Leah Strauch, Linda Sweet, Hayley Scott, Amanda Müller

**Affiliations:** 1 College of Medicine and Public Health, Flinders University, Adelaide, Australia; 2 School of Nursing and Midwifery, Deakin University and Western Health Partnership, 221 Burwood Hwy, Burwood, VIC 3125 Australia; 3 College of Nursing and Health Sciences, Flinders University, Adelaide, Australia

**Keywords:** Breastfeeding, Expressing, Support, Breastmilk feeding, Exclusive expression

## Abstract

**Background:**

The World Health Organization recommends that infants be exclusively breastfed until the age of six months. Breastfeeding is generally understood to mean the provision of human breastmilk to the infant by direct feeding at the breast, and interventions aimed at supporting exclusive breastfeeding are therefore targeted at this activity. However, breastfeeding is actually an umbrella term covering the provision of breastmilk to an infant by any means. Our population of interest is mothers who exclusively feed their infants indirectly using expressed breastmilk. Some research suggests that any expressing, and exclusively expressing in particular, can be a risk factor for early cessation of exclusive breastmilk provision, so we were interested to identify whether any specific support existed for exclusively expressing mothers outside of the context of premature infants and the Neonatal Intensive Care Unit setting.

**Methods:**

A scoping review following the Joanna Briggs Institute approach was used to explore the phenomenon of formal and informal supports in the community for exclusively expressing mothers. Searches were run across academic databases and of government websites and infant feeding support organisations. Finally, an informal internet search was run using a simple search string.

**Results:**

On analysis of results, there were no studies or articles that met the search criteria. An informal internet search linked us directly with websites and blogs that could be considered a form of support intervention. These informal results suggest that support material or programs could possibly exist in other modalities but we cannot find them in the context of this type of scoping review.

**Conclusions:**

The results of the search corroborated what we had suspected – that exclusively expressing mothers are not specifically supported by usual channels for new parents and that it is also difficult to find acknowledgement that exclusive expression exists.

The absence of results demonstrates the relevance of this study: exclusively expressing mothers are an under-served population. If we wish to strive towards achievement of World Health Organization breastfeeding goals, exclusively expressing mothers require targeted support to assist in their infant feeding experience, and there is little formal evidence of it currently being provided.

## Background

The World Health Organization (WHO) recommend that infants be exclusively breastfed until the age of 6 months [1]. The general public assumption about the term ‘breastfeeding’ is the provision of human breastmilk to the infant by direct feeding at the breast. This does not consider situations where mothers use breastmilk expression. Indeed, the WHO definition of ‘exclusive breastfeeding’ specifically states that this feeding practice requires that the infant receive ‘breast milk (including milk expressed or from a wet nurse)’ [2]. In reality, mothers who exclusively feed their infants breastmilk fall into three main categories: direct feeding only, a combination of direct feeding and expressing, and exclusively expressing only. The fact that the existence of these categories is not widely understood or precisely quantified is evident in the questions and manner in which results are published in infant feeding surveys, such as in the United States of America [3], United Kingdom [4], and Australia [5]. Furthermore, the Australian Medical Association discusses only breastfeeding and formula feeding in its position statement on infant health [6], with no reference to expressing generally. These examples demonstrate ways in which both expressing and exclusive expressing is subsumed under the umbrella term of ‘breastfeeding’, even though the actual experience of expressing and feeding expressed milk is vastly different from at-breast feeding for both mother and baby.

We hypothesise that the language used in this domain has emerged because of the infant-focused nature of most work – ‘breastfeeding’ as a proxy for ‘breastmilk-fed’ – when the outcome of interest is what the infant is consuming rather than how the substance has been produced or what the mother’s actions have been. Whilst we might prefer to use ‘breastfeeding’ solely in terms of the lay understanding, we must respect the context in which we work and the historical definitions in the infant feeding space. Therefore, in order to clearly identify and distinguish our population of interest, we will be using the terms ‘direct feeding’ to represent the provision of breastmilk directly from the breast into the infant’s mouth, and ‘breastmilk expression’ to represent the provision of breastmilk indirectly to an infant through the expression of breastmilk using an intermediary measure, such as mechanical pumping or manual extraction, and delivery to the infant via an infant feeding device such as bottle and teat.

As mentioned previously, there is a subset of mothers who exclusively feed their children expressed breastmilk, and never direct feed at the breast. The reasons for this can involve child or maternal physical health (for example an infant with a cleft palate, or maternal nipple trauma), maternal mental health (past trauma, anxiety/shame over breastfeeding), breast refusal, or environmental factors such as the requirements of other children or employment. These women are our population of interest and are our ‘exclusively expressing mothers’. The limited data available suggests that this group may include a substantial portion of parents, with studies indicating exclusive expressers to be between 5 and 22% of the total breastmilk-fed cohort [7–10].

It is common for the unique experience of exclusively expressing mothers to not be mentioned or considered in studies investigating the topic of infant feeding, particularly when there may be more striking comparisons to be made between the provision of breastmilk and of breastmilk substitutes such as artificial formula. Exclusive expressers are therefore somewhat hidden, both in the literature and in reality, as they deal with pumping around the clock in order to maintain supply, on top of all of the other responsibilities that come with having a new baby.

There are mixed results when considering the impact of expressing breastmilk on the duration of any (not exclusive) breastmilk feeding amongst all infants. Some studies identify that the introduction of expressed breastmilk into the infant’s feeding experience (either expressed only or a mix of expressed and direct) is a risk factor for earlier cessation of breastmilk feeding when compared with infants who receive breastmilk only directly at the breast [8, 11, 12]. Another study suggested the opposite – that introduction of expressed breastmilk meant a mother was less likely to discontinue breastmilk feeding before 6 months than a mother who only ever provided breastmilk directly – but did not compare the category of women who only ever expressed breastmilk- [13]. The outcomes of these studies did not stratify their participants into those who exclusively breastmilk-fed their infants, and therefore included formula supplementation. A single study which specifically considered exclusive expressing for the exclusively breastmilk-fed child identified that if a mother made it to 3 months post-partum exclusively expressing, she was at no higher risk of ceasing exclusive breastmilk feeding than her direct feeding counterparts, although she was more likely to cease breastmilk feeding in general. This study unfortunately was unable to include infants who were weaned prior to 3 months of age, and therefore could not comment on the risk of early cessation in the first months of an infant’s life [7, 8].

In our initial perusal of the breastfeeding literature, we found that when expression was the primary focus of the paper, it generally dealt with the initiation of expression or was focused on Neonatal Intensive Care Unit (NICU) experience. Studies and investigations such as these have mostly focused on education around breastmilk expression and the prevalence of lactation initiation. This is understandable, because the majority of women who need to express for a premature infant often have an ultimate goal to direct feed once their infant is able [14]. However, we did not find any literature on how to maintain full-time exclusive expression in situations where this will be the ongoing primary method of feeding. As noted above, there is a common thread that: a) any use of expressed breastmilk instead of direct feeding is suggested to be a risk factor to early cessation of exclusive breastfeeding (early being considered < 6 months duration); and b) that exclusive expression specifically is a risk factor to early cessation. A 2016 study [15] that included women who either aimed to express, or currently did express, in any way (casually through to exclusively) identified that women who express can be emotionally burdened, and this is accompanied by the physical and mental fatigue from the extra tasks of expressing and its associated management. Corroborating this is other qualitative work that suggests the time required for implementation of exclusive expressing is incompatible with full-time employment [3].

This limited literature is evidence of the paucity of research that examines how women can be supported to maintain exclusive breastmilk provision. If the WHO target of 6 months is to be met, this group of women need to be identified within the community and appropriate, tailored support provided. The needs of an exclusively expressing mother will be different to those of a direct feeding mother, and existing mechanisms of support may not translate across the two groups. We were interested in understanding what, if any, support programs, networks, education, or promotion, whether formal or informal, might be actively targeting exclusive expressers. Support could be practical or emotional, regarding the management of pumping equipment, the shared experience of expressing, schedules for pumping and volumes for feeding – anything that prioritises the exclusively expressing mother and specifically seeks to assist her.

A preliminary search for previous reviews on topics aligned to exclusive expressing was conducted in the Johanna Briggs Institute database and the Cochrane Library. Systematic reviews were found on the following complementary topics:

- Prevalence and outcomes of breast milk expressing in women with healthy term infants [16]
- Methods of milk expression for lactating women [17]
- Structured versus non-structured breastfeeding programmes to support the initiation and duration of exclusive breastfeeding in acute and primary healthcare settings [18]

Each of these reviews captured an element of the topic of interest but did not answer the question of whether support networks and programs exist for mothers who exclusively express. This scoping review therefore aimed to collate any available literature regarding exclusive expressing and forms of formal and informal support for the exclusive expressing population. The review question was “For mothers who exclusively express their breastmilk, what, if any, formal or informal supports are available in the community?” We focused on supports in the community setting, rather than hospitals and NICUs where expression is a short-term solution and direct feeding is usually the final goal.

## Methods

A scoping review following the Joanna Briggs Institute (JBI) approach [19] was used to explore the phenomenon of formal and informal supports in the community for exclusive expressers. The types of studies and articles sought were those with participants who were exclusively expressing breastmilk in a non-hospital setting and who had interacted with a support network or program of any type that was directed at them specifically as exclusive expressers. This could be a face-to-face program initiated by hospitals or other health care providers or community peer-based programs. Alternatively, formal online or telephone support through a health service, or an informal network of other expressing mothers would also qualify. The inclusion and exclusion criteria are shown in Table 1.

**Table 1:** Inclusion and exclusion criteria

Inclusion	Exclusion
Population is wholly or primarily exclusively expressing mothers	Population is any mothers of infants with no focus on expression or exclusive expression
Intervention is a support program or network targeted to exclusive expression	Intervention is focused on initiation of expression
Population is located in a community (non-hospital) setting	Population is located in a hospital/NICU setting
	Population is solely focused on premature infants

The services of a medical research librarian were enlisted to guide and validate the search strategy for this review. In summary, after an initial brainstorm of relevant terms, a preliminary limited search of MEDLINE, CINAHL, and EmCare was undertaken to identify index terms and subject headings, which allowed development of a logic grid for a text word search. Customised searches to suit syntax and index terms were run across MEDLINE, CINAHL, EmCare, Scopus, PubMed, Cochrane, and JBI. An example of a search strategy used is shown in Table 2 with the complete search for CINAHL provided.

**Table 2:** Example of search strategy CINAHL

Search ID#	Search Terms
S9.	S3 AND S8
S8.	S4 OR S5 OR S6 OR S7
S7.	TI (((educat* or knowledge or online or peer or communit* or social* or intervention* or program*) N4 support)) AND AB (((educat* or knowledge or online or peer or communit* or social* or intervention* or program*) N4 support))
S6.	(MH “Support, Psychosocial”)
S5.	(MH “Health Promotion”)
S4.	(MH “Health Education”)
S3.	S1 OR S2
S2.	TI (((express* or pump* or indirect*) N3 (milk* or breastmilk*))) OR AB (((express* or pump* or indirect*) N3 (milk* or breastmilk*)))
S1.	(MH “Milk Expression”)

A targeted search was undertaken of Australian national, state, and territory health websites, the Australian Breastfeeding Association and major Anglosphere (United Kingdom, New Zealand, United States of America, and Canada) government websites, and infant feeding support organisations. A further informal online search was made across Google and Bing using a simple search string to identify potential internet-based supports. These formal, targeted and informal searches were each undertaken by one researcher (LSt). In these searches no restriction was placed on publication date, while publication language was restricted to English. This was was in line with our geography of interest given our expectation of different experiences for mothers across a wider range of cultural backgrounds.

## Results

**Fig. 1:**
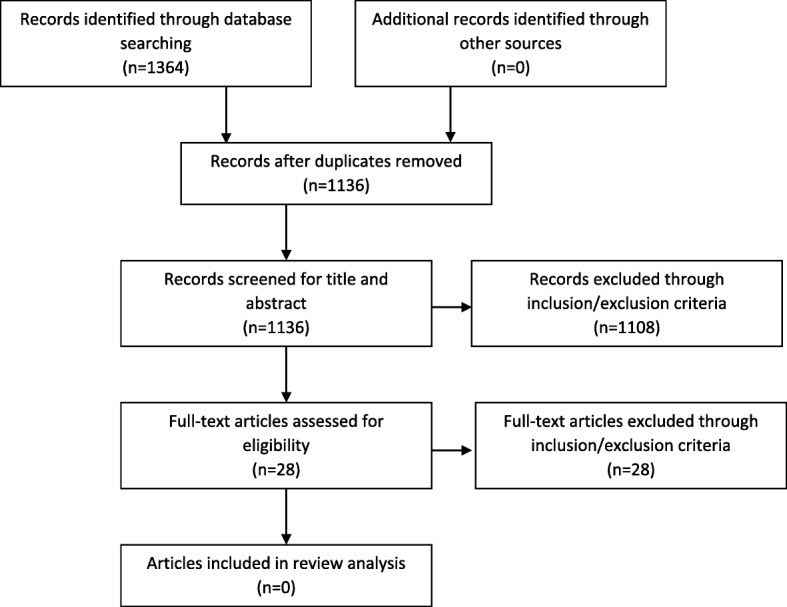
PRISMA diagram illustrating literature search

Following the extensive search strategy, there were no studies or articles that met the search criteria. Whilst there were articles considered during the review process, none focused on support systems for mothers who are exclusively expressing breastmilk. This is shown in Fig. 1.

Through the database searches, no paper was identified that considered the experience of exclusive expressing in the community; however, there were some linked to the experience of families with premature infants, and the initiation of breastmilk expression in the NICU.

Within the directed search, there was a single result that specifically mentioned ‘exclusive expressing’ [20], and a small number that ambiguously referred to potential mothers who expressed ‘full time’ [21] or for ‘a long time’ [22]. No clear evidence of available support was noted, either through the websites themselves or referenced in their text.

The lack of results from the formal search was not surprising based on the previous reading we had completed in this area. However, we were also aware of the existence of at least one type of specifically targeted support: peer support group run via social media. For this reason, a further informal internet search was undertaken by one researcher (LSt) using a simple search string in Google in ‘incognito’ mode. Incognito mode was used as a means to limit the impact of previous search history of the authors on new results. This provided a snapshot into some specific resources that have been published online with exclusive expressers as the target audience. Results included one-way information pages on websites from private authors (doctors, lactation consultants, doulas and interested parents) and pump manufacturers as well as two-way discussions on hosted forums on parenting websites. Some of the top results with a description of their content are listed in Table 3.

**Table 3:** Sample of search results on ‘informal’ internet search

Website	Article title	Author type	Description
Essential Baby [23]	Who else exclusively feeds expressed breast milk?	Parents (online forum)	Two-way communication. Single discussion on forum. Discussion thread of exclusively expressing mothers; no notes of support but anecdotes of being told to see lactation consultant
Belly Belly [24]	Exclusively pumping breast milk	Organisation (no specific author listed), possibly sponsored	One-way information. Single article on website. List of sensible advice to assist exclusively expressing mothers, and links to supportive websites, blog and books. Also specific promotion of Spectra pump.
KellyMom [25]	Exclusive pumping	Individual parent, reviewed by lactation consultant	One-way information. Single article on website. Specific advice and tips to manage expressing in different situations.
Spectra Baby [26]	Exclusive pumping: 9 things you need to know	Product manufacturer	One-way information. Single article on website. Clear advice on optimising use of pump to express.
Baby Hints and Tips [27]	Exhausted with exclusive expressing	Individual parents	One-way information. Single article/discussion. Summary of message board asking for help – responses both positive and negative regarding
Exclusive Pumping [28]		Individual parent	One-way information. Entire website dedicated to exclusive pumping and managing it.
Baby Centre [29]	Pros and cons of exclusively expressing	Individual parents	Two-way communication. Single discussion on parenting forum. Frank discussion between exclusively expressing mothers of the pros and cons of the practice.

By utilising an informal internet search, we were able to demonstrate the existence of web-based resources created by, and for, exclusively expressing mothers. The results of this search were for the support mechanisms themselves, rather than a description of them, which meant that a limitation of this search was that it only returned evidence of internet pages or blogs, and not support across a broader range of online outlets.

## Discussion

The initial goal of this scoping review was to identify formal and informal supports offered for exclusive expressers and, where available, contrast any identified outcomes of support provision, such as duration of exclusive expressing or improvement in the maternal experience. This was identified as a relevant area for a formal search due to a noted lack of recognition of exclusively expressing mothers as a unique parent group within broader breastfeeding literature.

Ideally, we hoped to uncover comparable work to that of direct breastfeeding, such as an analysis of the impact of support programs in different modalities. In the context of improving uptake or extending duration of exclusive direct feeding, enough programs have been implemented that effectiveness of different variables can be compared such as the type of provider, modality of provision, and whether the intervention is real time or asynchronous [18, 30–32]. We identified no such ‘ideal’ work for exclusive expressers. What we did find, in the informal search, was evidence of several types of online resources available for parents who seek them out. These included one-way (blog, article), asynchronous (message board), and real-time (live messaging) support by both professionals and lay-persons. It is difficult to gauge what impact these might have for parents; in the case of the message board, a query regarding “who else exclusively feeds expressed breast milk” attracted 9 replies (with a one duplicate poster) over the course of 8 days [23].

The exclusion of material aimed at parents of preterm infants was important because the primary goal would be lactation maintenance until direct feeding is possible, which is different to long-term planned exclusive expression. However, it effectively removed most studies, articles, or information that we identified focused around education and support for exclusive expression. For example, a systematic review that specifically looked at methods of milk expression [17] contained references to studies comparing the improved experience of mothers when they are provided with specific instruction around expression [33, 34], but it is defined by its focus both on initiation and preterm infants, and thus was excluded.

The results of the search ultimately corroborated what we had initially suspected – that exclusively expressing mothers are not specifically supported by usual channels for new parents (e.g. government health organisations, infant feeding organisations), and that it is also almost impossible to find acknowledgement that exclusive expression exists. Of the directed searches, only the Australian Breastfeeding Association included a small amount of information in their internet content identifying exclusive expression, its difficulty, and that exclusively expressing mothers deserve support [20], although potential forms of support were not described.

At the same time, we know that mothers who exclusively express are a group who want to be heard. A notable outcome from one study working with women who were not able to direct feed was that every single woman who was identified as part of the target population volunteered to take part in that study [35]. Also, this same study highlights the opposite of what we hoped to find – there was an articulated lack of support for women who give up direct breastfeeding and a need to make the decision between exclusive expressing or formula. These authors found that exclusive expressing mothers want help: one of the quoted participants poignantly describes wishing for ‘just one [resource] about not breastfeeding’ among the plethora of information about breastfeeding [35]. Even more starkly, the outcome of a lack of support is clearly enunciated when considering what might happen with the next baby – with one participant stating ‘I shall never express milk again’ [35]. This work clearly found that women who exclusively express needed special and specific support that they were not receiving from the health professions. This scoping review highlights the hidden nature of exclusive expressers and the absence of any support programs to protect and promote the long-term provision of human breastmilk for babies who are not fed directly at the breast.

## Conclusions

This scoping review was undertaken with a view to understanding whether the exclusively expressing population was provided with specific support and the effect of different types of support to help mothers maintain longer-term exclusive expression. The unique physical and emotional requirements of exclusively expressing mothers separate them from those who supplement direct feeding with intermittent expression, or those who use formula milk.

Academic, formal, and directed internet searches yielded no results discussing targeted support programs or networks focused on exclusive expressers. An informal internet search linked us directly with websites and blogs that could be considered as a form of support intervention. These informal results suggest that support material or programs could possibly exist in other modalities (telephone, face-to-face) but we have no way of finding them in the context of this type of scoping review.

The absence of results demonstrates the relevance of this study: exclusive expressers are an under-served population making up a small but significant portion of the total breastfeeding cohort. If we wish to strive towards universal achievement of WHO breastfeeding goals, exclusive expressers require targeted support to assist in their infant feeding experience, and there is little formal evidence of it currently being provided.

## Abbreviations

NICU: Neonatal Intensive Care Unit
WHO: World Health Organization
